# Type 2 Diabetes Research Yield, 1951-2012: Bibliometrics Analysis and Density-Equalizing Mapping

**DOI:** 10.1371/journal.pone.0133009

**Published:** 2015-07-24

**Authors:** Fiona Geaney, Cristian Scutaru, Clare Kelly, Ronan W. Glynn, Ivan J. Perry

**Affiliations:** 1 Department of Epidemiology and Public Health, University College Cork, Cork, Ireland; 2 Charité-Universitätsmedizin Berlin, Free University Berlin and Humboldt-University Berlin, Berlin, Germany; 3 Department of Public Health, HSE Eastern Region, Dr Steevens' Hospital, Dublin 8, Ireland; Hellas, GREECE

## Abstract

The objective of this paper is to provide a detailed evaluation of type 2 diabetes mellitus research output from 1951-2012, using large-scale data analysis, bibliometric indicators and density-equalizing mapping. Data were retrieved from the Science Citation Index Expanded database, one of the seven curated databases within Web of Science. Using Boolean operators "OR", "AND" and "NOT", a search strategy was developed to estimate the total number of published items. Only studies with an English abstract were eligible. Type 1 diabetes and gestational diabetes items were excluded. Specific software developed for the database analysed the data. Information including titles, authors’ affiliations and publication years were extracted from all files and exported to excel. Density-equalizing mapping was conducted as described by Groenberg-Kloft et al, 2008. A total of 24,783 items were published and cited 476,002 times. The greatest number of outputs were published in 2010 (n=2,139). The United States contributed 28.8% to the overall output, followed by the United Kingdom (8.2%) and Japan (7.7%). Bilateral cooperation was most common between the United States and United Kingdom (n=237). Harvard University produced 2% of all publications, followed by the University of California (1.1%). The leading journals were Diabetes, Diabetologia and Diabetes Care and they contributed 9.3%, 7.3% and 4.0% of the research yield, respectively. In conclusion, the volume of research is rising in parallel with the increasing global burden of disease due to type 2 diabetes mellitus. Bibliometrics analysis provides useful information to scientists and funding agencies involved in the development and implementation of research strategies to address global health issues.

## Introduction

The burden associated with type 2 diabetes mellitus (T2DM) continues to escalate in developed and developing countries [1–4]. The global prevalence is expected to rise from 6.4% (285 million) in 2010 to 7.7% (439 million) in 2030 among adults aged 20–79 years [5]. The economic cost of T2DM in the United Kingdom (UK) in 2010/2011 was £8.8 billion for direct costs and £13 billion for indirect costs, and this is forecasted to increase to £15.1 billion for direct costs and £20.5 billion for indirect costs by 2035/2036 [6].

Bibliometrics is a useful method to evaluate trends in research activity over time and to inform future policy. Findings from bibliometrics studies have played a fundamental role in decision making regarding policy formation and the prioritisation of resources for public health challenges i.e. the Research Assessment Exercise in the UK [7]. Bibliometrics studies have also been conducted to examine the trends in medical research output for gastroenterology [8], infectious diseases [9, 10], microbiology [11], oncology [12, 13], otolaryngology [14], respiratory medicine [15], surgery [16] and public health [17–19].

Few bibliometrics studies relating to diabetes exist [20–23]. These previous studies focused on individual countries including Nigeria, Thailand, Argentina and China. Studies do not exist relating to the global research output for T2DM only. Given its associated disease burden, there is a need to conduct a bibliometrics study on the published literature relating to T2DM to investigate if the increasing global prevalence of this disease is reflected in trends in the literature. Therefore, the aim of this study was to provide a detailed evaluation of the T2DM research output from 1951–2012 using a specifically developed software to quantitatively analyse data from the Web of Science, Science Citation Index Expanded (WOS SCI-Expanded) database in terms of (1) numbers of published items and citations (2) country specific publications (3) international collaboration and (4) publications by journals and subject areas.

## Materials and Methods

Data were retrieved in February 2013 from the WOS SCI-Expanded database produced by the Thomson Reuters. This citation database is one of seven databases within the Web of Science (WOS). It is a multidisciplinary index of the scientific journal literature. This human curated database indexes over 8,500 journals across 150 disciplines and contains all cited references from the indexed articles. It provides complete reference data for all publications.

With the use of Boolean operators "OR", "AND" and "NOT", the following search query was developed to estimate the total number of published items related to type 2 diabetes; ((NIDDM) OR (Maturity-Onset Diabetes) OR (Diabetes Mellitus Noninsulin-Dependent) OR (Diabetes Mellitus Adult-Onset) OR (Adult-Onset Diabetes Mellitus) OR (Diabetes Mellitus Adult Onset) OR (Diabetes Mellitus Ketosis-Resistant) OR (Diabetes Mellitus Ketosis Resistant) OR (Ketosis-Resistant Diabetes Mellitus) OR (Diabetes Mellitus Maturity-Onset) OR (Diabetes Mellitus Maturity Onset) OR (Diabetes Mellitus Non-Insulin Dependent) OR (Diabetes Mellitus Non-Insulin-Dependent) OR (Non-Insulin-Dependent Diabetes Mellitus) OR (Diabetes Mellitus Noninsulin Dependent) OR (Diabetes Mellitus Slow-Onset) OR (Diabetes Mellitus Slow Onset) OR (Slow-Onset Diabetes Mellitus) OR (Diabetes Mellitus Stable) OR (Stable Diabetes Mellitus) OR (Diabetes Mellitus Type II) OR (Diabetes Mellitus Type 2) OR (Maturity-Onset Diabetes Mellitus) OR (Maturity Onset Diabetes Mellitus) OR (MODY) OR (Type 2 Diabetes Mellitus) OR (Noninsulin-Dependent Diabetes Mellitus)). Published items that included type 1 diabetes and gestational diabetes were also searched and then excluded from the analysis (S1 Appendix).

Certain limits were also applied to the search query (S1 Appendix). The time period under study was 1951–2012. The search was conducted in February 2013 and therefore, 2013 was eliminated from the analysis as complete data for that year was unavailable. The search included all document types including original articles, reviews, letters and editorials. All published items also had to provide an English abstract to be eligible for the inclusion criteria.

The results of the search were reviewed once the query was performed. To export all necessary information from all articles, plain text files and full records were selected in the search results page. The topic of each article was also selected rather than the title as the topic search also examines the keywords and the abstract too.

Specific software developed by the Charité University in Berlin was used to quantitatively analyse data from the WOS SCI-Expanded database (S2 Appendix). Once the search criteria was entered, the data was exported from the database and each data item was downloaded and contained in a ‘data block’ as text files. All data blocks were tagged and the software separated the provided information on the content of the block including AU = authors, TI = title, PY = publication year and AF = affiliation. Initially, the software read each tag and the linked data and saved it to a Microsoft Access database. The software highlighted any invalid addresses (usually due to the use of acronyms) and asked the user to identify the correct addresses. The user then located all of the correct addresses with the use of Google Scholar. Data on institutions and authors were reviewed using the same method. There was no missing data regarding addresses, institutions or authors.

The data was then exported to a Microsoft Excel database for descriptive analysis. Published items were examined using the citation report method [24, 25]. The number of citations per year and the average number of citations per item were investigated. The average number of citations per item was calculated based on the number of citations divided by the number of published items found.

Density equalizing mapping was employed as described by Groeneberg-Kloft et al in 2008 [26]. All countries responsible for publishing the literature were scaled according to different variables of interest including the number of published items and the average number of citations per item for each country. Calculations were based on Gaster and Newman's algorithm [27]. From the concepts of elementary physics, these calculations incorporated a diffusion equation in the Fourier domain, which enabled variable resolution by tracking moving boundaries [27]. Colour coded legends were presented to explain the scaling of the maps. For the map relating to the average number of citations per item for each country, a threshold excluded countries with less than 30 published items to improve clarity.

Cooperation analysis was conducted based on the author’s affiliations to examine bilateral and multilateral cooperation between countries and between institutions on T2DM research. The cooperation network was developed by examining all combinations of the countries and of the institutions that registered international cooperation’s on at least 10 items from 1951 to 2012. The data was then saved to a two-dimensional table. From the table, radar charts were designed and the software created a density-equalising map to illustrate the collaboration between countries. The subject categories for the published items were also analysed.

The journals that published the items relating to T2DM were investigated according to the number of published items, number of citations, average number of citations per item, impact factor and the Eigenfactor scores. The impact factor and Eigenfactor scores were extracted from the Thomson Reuters Journal Citation Reports (JCR). The impact factor score is calculated based on the numerator (the number of citations in the current year to items published in the previous two years) and the denominator (the number of substantive articles and reviews published in the same two years) [28]. The Eigenfactor endeavours to rate the influence of journals. The Eigenfactor score is calculated based on a complex algorithm that corresponds to a model of research where individuals trail a sequence of citations as they move from journal to journal. The score considers the quantity of citations and their "quality" by assigning weights to the source of the citations. The Eigenfactor scores are scaled to ensure that the sum of the scores of all journals listed in the Thomson’s JCR is 100. The impact factor scores were available for 2012. The Eigenfactor scores were available for 2011 only.

## Results

### Publications by year

**Fig 1:**
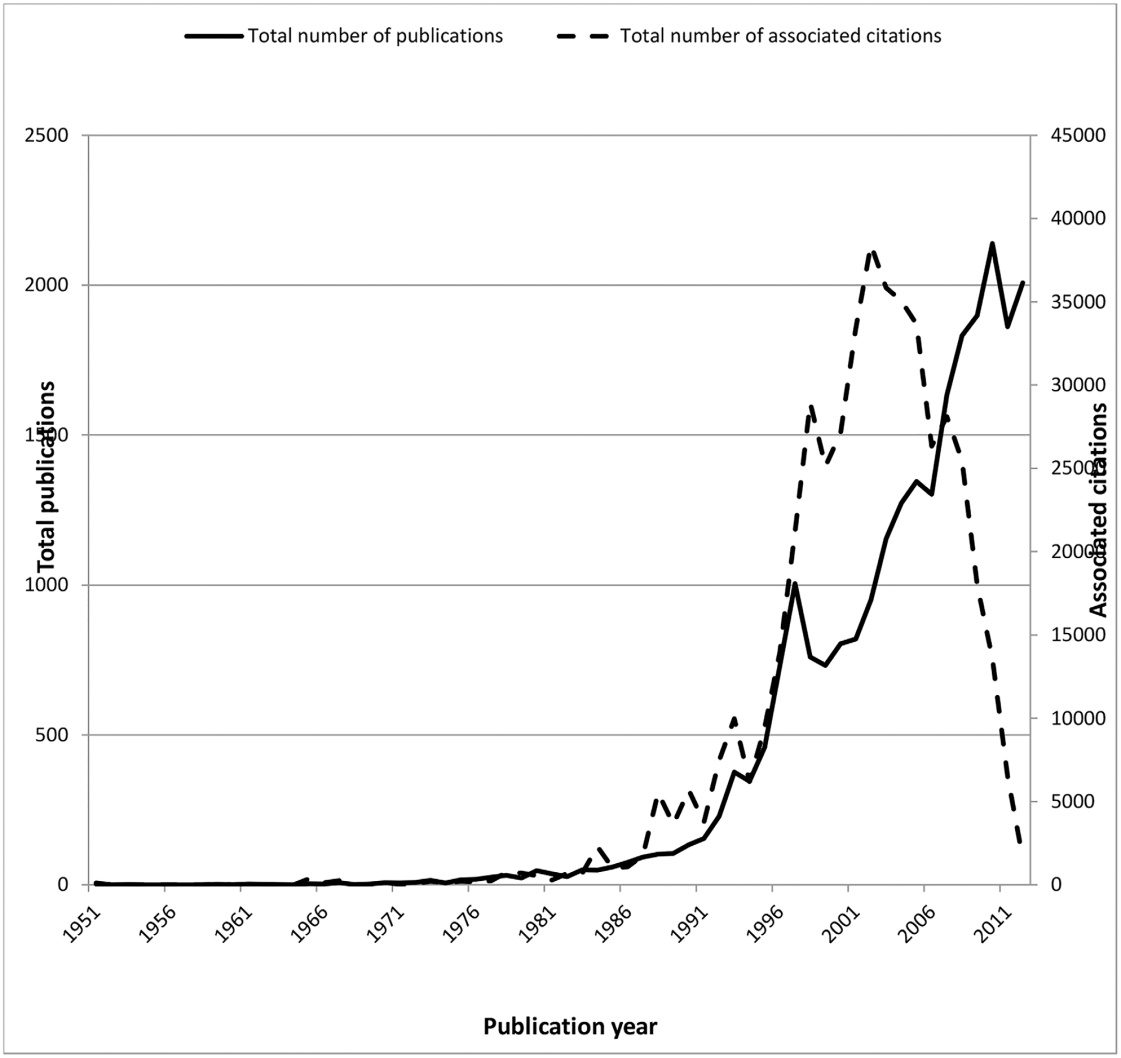
Distribution of T2DM publications and associated citations in the WOS SCI-Expanded database from 1951–2012 (—total number of publications,—total number of associated citations).

During the time period 1951–2012, 25,271 items relating to T2DM were published and indexed in the WOS SCI-Expanded database. A total of 488 published items were excluded before analysis due to inadequate information i.e. missing data on authors’ affiliations. Overall, 24,783 items were analysed and these items were cited 476,002 times. As expected, a strong correlation was observed between the number of citations and the number of publications (Fig 1). Studies relating to T2DM were not recorded in the WOS SCI-Expanded database until 1951 (n = 3). The frequency of publications started to increase steadily in the late 1980’s (1983, n = 50) (Fig 1) but sharply increased in the 1990’s (1997, n = 1005) (Fig 1). The greatest number of outputs were published in 2010 (n = 2,139). Articles published in 2002 received more citations (n = 38,412) than the other years. From 2001–2012, a descending trend was observed for the average number of citations per item.

### Publications by country

**Fig 2:**
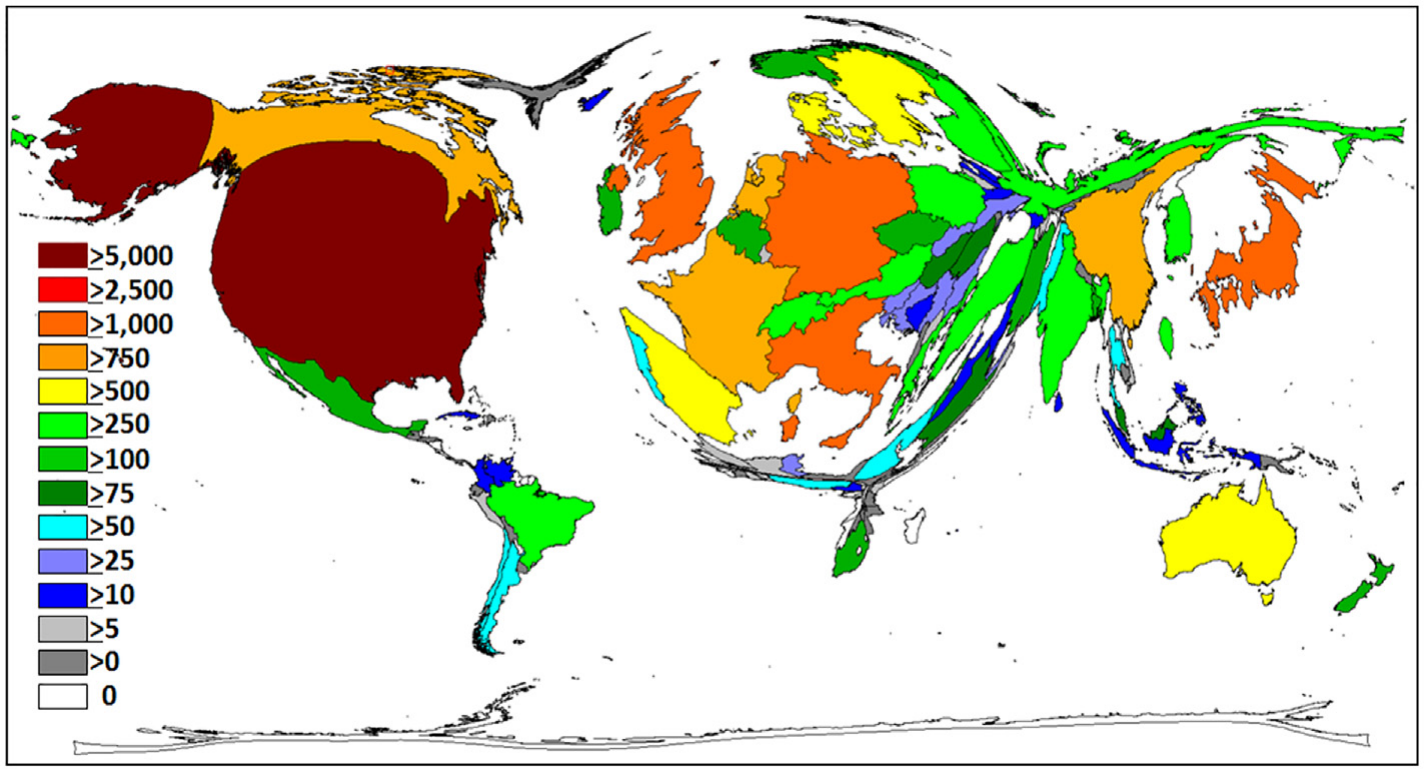
Density equalizing mapping, total output by country. Illustration of the total number of T2DM items, per country. The size of each country is scaled in proportion to the total number of publications. The colour coded legend shows the publication numbers.

**Fig 3:**
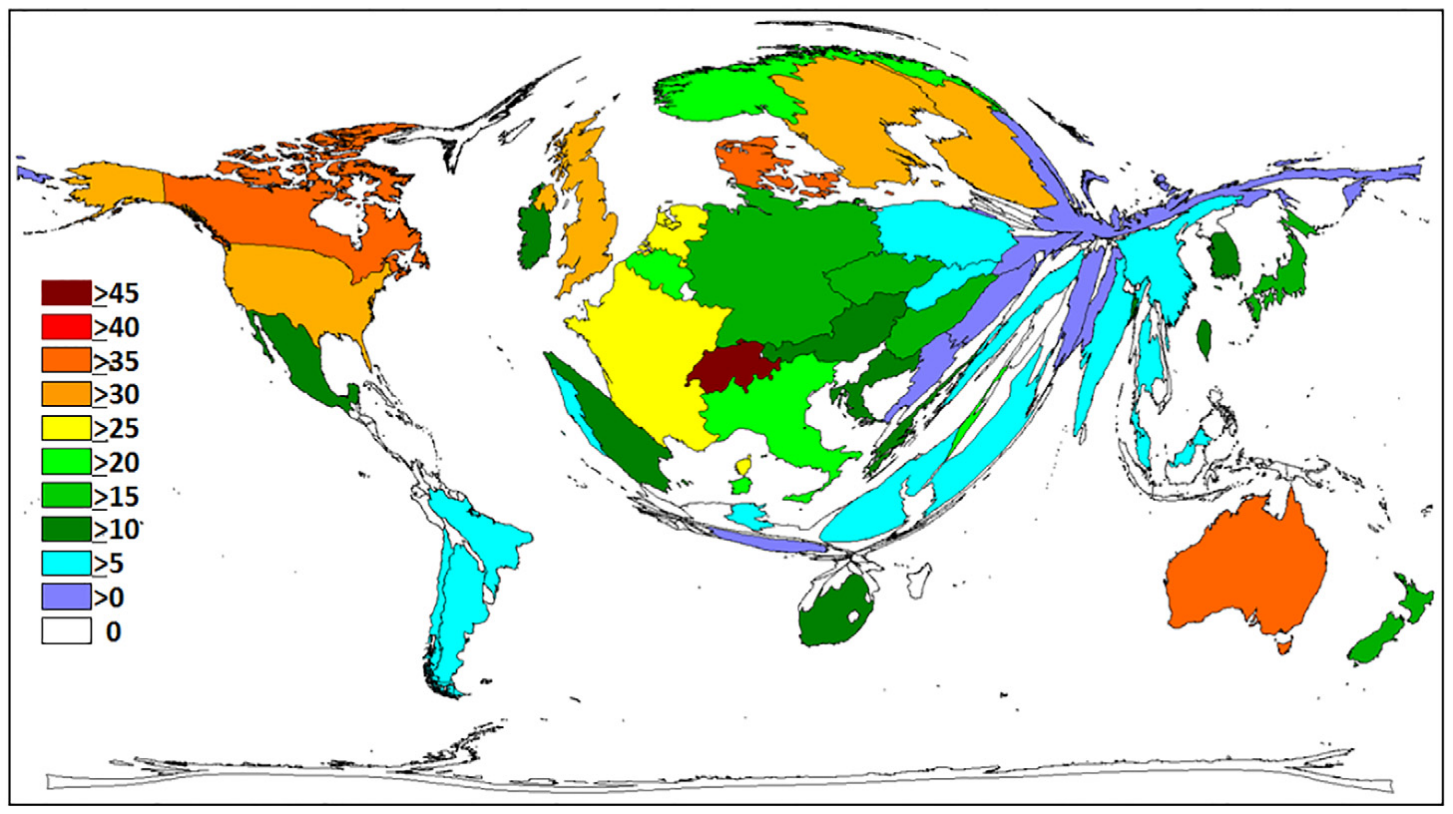
Density equalizing mapping, average number of citations per T2DM related item by country. The size of each country is scaled in proportion to the average number of citations per item. The colour coded legend shows the average number of citations per item. Threshold excludes countries with ≥30 items published.

A total of 129 countries contributed to the overall published output during the study period. The United States of America (USA) published the highest number of publications (n = 7,134) (Table 1). The density equalizing mapping in Fig 2 illustrates that a small number of countries were accountable for most of the output as the size of each country was scaled in proportion to the total number of publications. The USA contributed to 28.8% of the overall output, followed by the United Kingdom (UK) (8.2%), Japan (7.7%) and Germany (6.0%). The USA and the UK also received the greatest number of citations, respectively (n = 232,431; 67,715). Switzerland (>45) had the highest citation average per item (47.18) (Fig 3). Denmark, Australia and Canada recorded a citation average greater than 35, while countries like the UK, USA, Sweden and Finland received an average greater than 30.

**Table 1:** Leading 25 countries by number of T2DM publications and average citations per item, 1951–2012.

Rank	Country	No. of publications	Received citations	Average citations per item*
1	**United States**	7134	232431	32.58
2	**United Kingdom**	2031	67715	33.34
3	**Japan**	1917	30486	15.90
4	**Germany**	1497	28606	19.11
5	**Italy**	1359	30793	22.66
6	**France**	904	23152	25.61
7	**Netherlands**	826	21342	25.84
8	**Canada**	796	28355	35.62
9	**China**	790	7727	9.78
10	**Australia**	748	28701	38.37
11	**Spain**	726	10709	14.75
12	**Sweden**	622	19626	31.55
13	**Denmark**	535	20884	39.04
14	**India**	494	4637	9.387
15	**Turkey**	482	3801	7.89
16	**South Korea**	408	4902	12.01
17	**Brazil**	403	3286	8.15
18	**Finland**	388	11926	30.74
19	**Greece**	344	4426	12.87
20	**Switzerland**	315	14862	47.18
21	**Taiwan**	294	3386	11.52
22	**Austria**	277	3615	13.05
23	**Poland**	268	2551	9.52
24	**Russia**	263	626	2.38
25	**Israel**	237	5298	22.35

* Average citations per item = number of citations/ number of published items

### International collaboration

**Fig 4:**
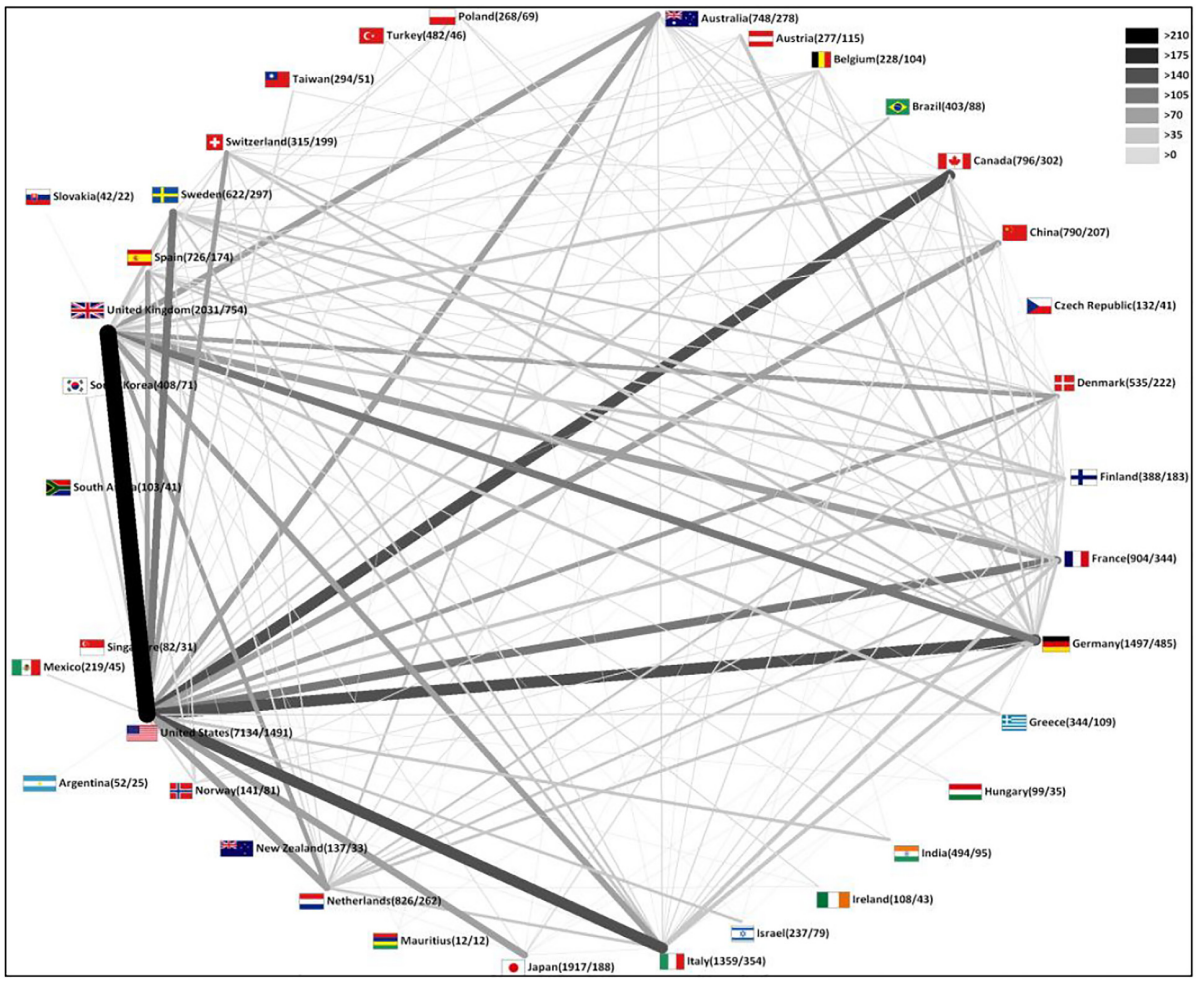
Radar chart of international collaboration density. Threshold ≥10 publications due to international collaborations.

Cooperation analysis was conducted to examine the international collaboration observed during the time period. International cooperation steadily increased from the early 1990's but the greatest numbers of cooperations for published items were indexed in 2010, with 367 cooperated items. Bilateral cooperation was the most common type of cooperation (n = 2,325 items), followed by trilateral cooperation (n = 440) and quadrilateral cooperations (n = 131). Bilateral cooperation was most frequent between the USA and the UK (n = 237), followed by the USA and Germany (n = 167) (Fig 4). The USA (n = 14) and the UK (n = 11) contributed to the majority of these bilateral cooperations (Table 2).

**Table 2:** Top 15 collaborating relationships, T2DM related items, 1951–2012.

Rank	Country 1	Country 2	Published items
**1**	United Kingdom	United States	237
**2**	Germany	United States	167
**3**	Canada	United States	165
**4**	Italy	United States	159
**5**	France	United States	119
**6**	Germany	United Kingdom	110
**7**	Sweden	United States	106
**8**	France	United Kingdom	105
**9**	Japan	United States	101
**10**	Netherlands	United States	100
**11**	Australia	United States	93
**11**	China	United States	93
**12**	Australia	United Kingdom	92
**13**	Italy	United Kingdom	87
**14**	Denmark	United States	83
**15**	Switzerland	United States	76

### Institutional collaboration

**Fig 5:**
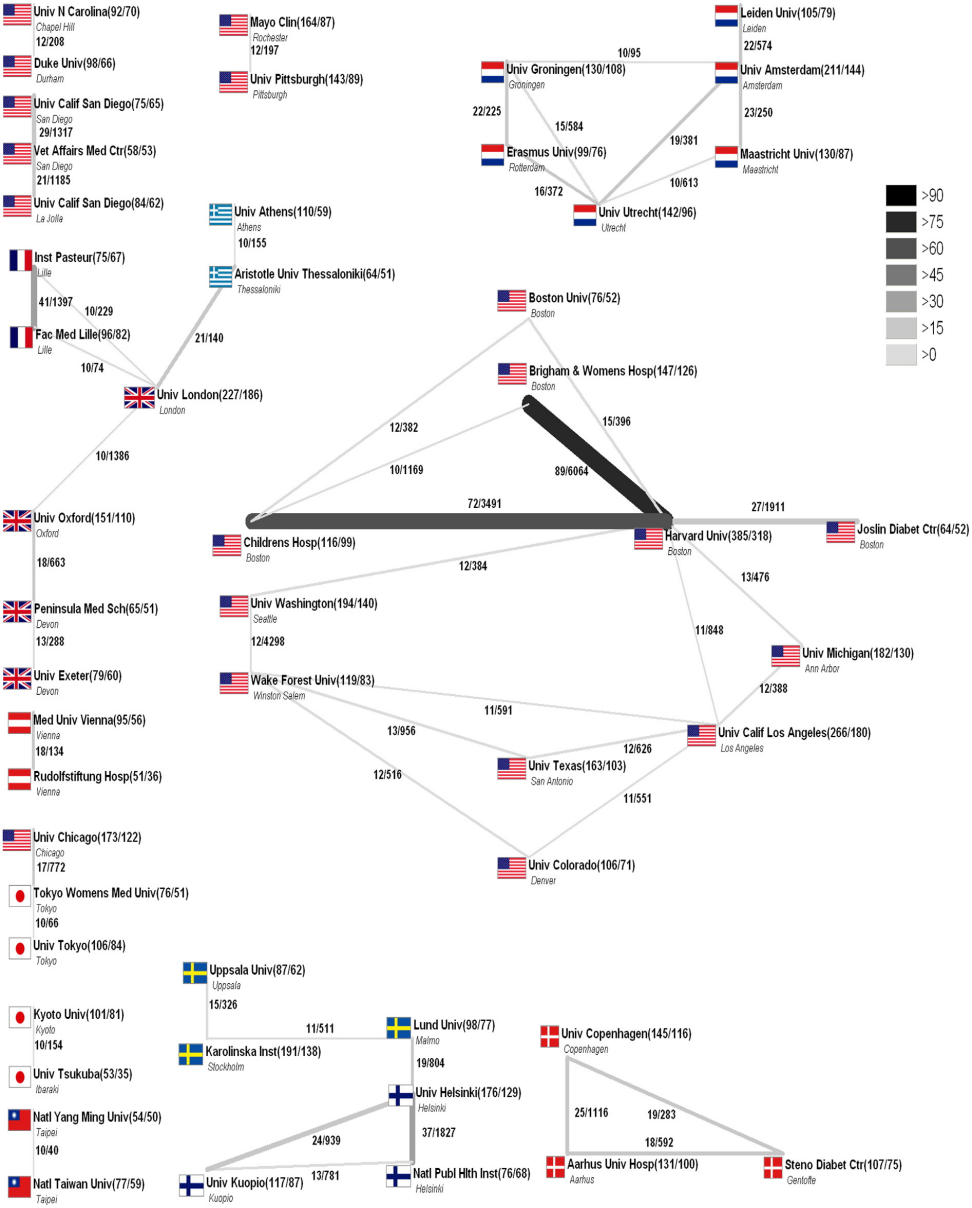
Radar chart of institutional collaboration density. Threshold ≥50 published items and >10 collaborations.

Harvard University in Boston produced 2% of the overall T2DM research output, followed by the University of California in Los Angeles (1.1%) and the University of London (0.92%). The majority of institutions collaborated with other institutions within the same country (Fig 5). Of the 285 articles published by Harvard University, 318 articles were in cooperation with another institution. Harvard University and the Children's Hospital in Boston created 72 articles in cooperation and these articles were cited 3491 times during the study period (Fig 5). International institutional collaboration was rare but was most common among the University of Chicago and Tokyo’s Women’s Medical University (n = 1, cited 772 times).

### Publications by journal

A total of 2,498 journals published at least one item related to T2DM from 1951–2012. Publications in this area were most frequent from three leading journals including Diabetes (n = 2303), Diabetologia (n = 1797) and Diabetes Care (n = 989) as seen in Table 3. These three journals represented 9.3%, 7.3% and 4.0% of the overall output, respectively. The leading 20 journals contributed 36.4% of the overall publication output (9031/24783) and the Eigenfactor scores ranged from 0–0.53 (Table 3).

**Table 3:** Leading 20 journals by T2DM-related published items.

Rank	Name	Published items	Citations	Impact factor	Eigenfactor
1	**DIABETES**	2303	40398	8.29	0.10
2	**DIABETOLOGIA**	1797	9977	6.84	0.06
3	**DIABETES CARE**	989	53339	8.09	0.11
4	**DIABETES RES CLIN PR**	481	4707	2.75	0.16
5	**DIABETIC MED**	408	7871	2.90	0.02
6	**METABOLISM**	286	4599	2.66	0.02
7	**CIRCULATION**	286	17890	14.74	0.34
8	**J CLIN ENDOCR METAB**	247	10278	5.97	0.13
9	**J HYPERTENS**	200	2705	4.02	0.03
10	**VALUE HEALTH**	184	102	2.19	0.01
11	**DIABETES OBES METAB**	178	1982	3.38	0.01
12	**ATHEROSCLEROSIS**	163	2200	3.79	0.05
13	**AM J CARDIOL**	155	3497	3.37	0.08
14	**INT J OBESITY**	135	3248	4.69	0.04
15	**J AM COLL CARDIOL**	131	2953	14.16	0.22
16	**LANCET**	122	13901	38.28	0.36
17	**CLIN RES CARDIOL**	120	67	2.95	0.00
18	**J DIABETES COMPLICAT**	114	1386	2.03	0.00
19	**EUR HEART J**	113	1290	10.48	0.11
20	**HORM METAB RES**	106	1701	2.19	0.01

Impact factor and Eigenfactor scores from Thomson Reuters JCR.

**Fig 6:**
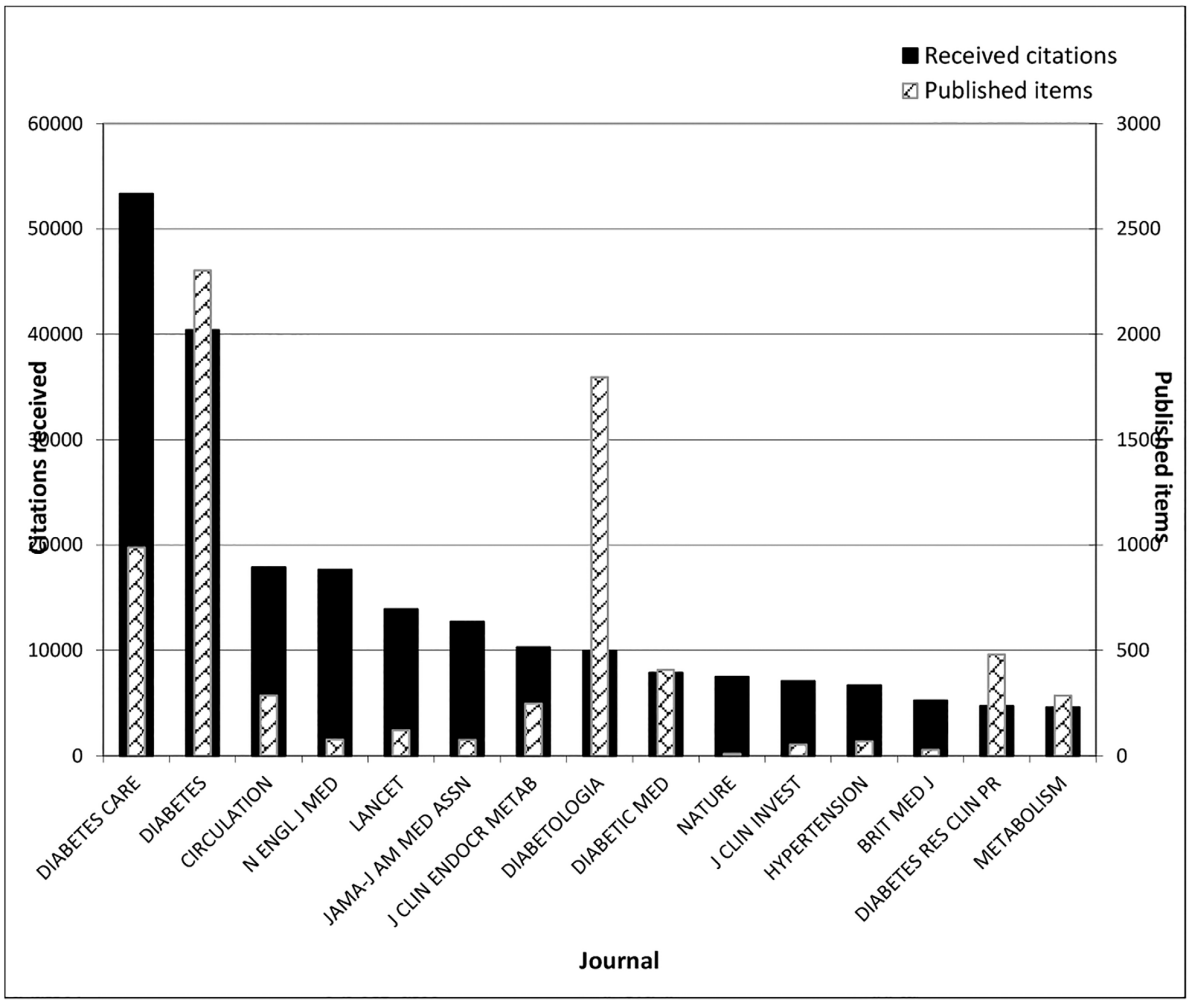
Leading 15 journals by T2DM-related published items and number of received citations, 1951–2012.

The ranking of journals differed when the numbers of citations per journal were compared. Diabetes Care (n = 53,339) received the highest number of citations followed by Diabetes (n = 40,398), Circulation (n = 17,890) and the New England Journal of Medicine (n = 17,653) (Fig 6). The New England Journal of Medicine received the greatest average number of citations per item 232.28 (17653/76) followed by the Journal of the American Medical Association (JAMA) (n = 169.64), Journal of Clinical Investigation (n = 133.81) and the Lancet (n = 113.94) (Table 4). The New England Journal of Medicine also reported the highest impact factor (n = 53.3). The principal subject categories of the extracted journals were ‘Endocrinology & Metabolism’ (39.6%), ‘Cardiovascular System & Cardiology’ (11.5%) and ‘General & Internal Medicine’ (11.4%). The Proceedings of the National Academy of Sciences of the USA of America received the highest Eigenfactor score, 1.60 (Table 4).

**Table 4:** Top 20 journals by average citations per T2DM item.

Rank	Name	Average citations per item*	Citations	Published items	Impact factor	Eigenfactor
1	**N ENGL J MED**	232.28	17653	76	53.3	0.66
2	**JAMA-J AM MED ASSOC**	169.64	12723	75	30.03	0.29
3	**J CLIN INVEST**	133.81	7092	53	13.07	0.22
4	**LANCET**	113.94	13901	122	38.28	0.36
5	**HYPERTENSION**	98.28	6683	68	6.21	0.07
6	**P NATL ACAD SCI USA**	91.65	3391	37	9.68	1.60
7	**CIRCULATION**	62.55	17890	286	14.74	0.34
8	**GASTROENTEROLOGY**	61.07	2687	44	11.68	0.16
9	**DIABETES CARE**	53.93	53339	989	8.09	0.11
10	**TRENDS ENDOCRIN MET**	53.83	1615	30	8.12	0.02
11	**AM J KIDNEY DIS**	50.84	2593	51	5.43	0.04
12	**HEPATOLOGY**	49.06	1570	32	11.67	0.12
13	**ENDOCRINOLOGY**	45.79	1786	39	4.46	0.09
14	**ARCH INTERN MED**	45.27	4165	92	11.46	0.12
15	**AM J CLIN NUTR**	45.01	4051	90	6.67	0.09
16	**AM J EPIDEMIOL**	42.88	1372	32	5.22	0.07
17	**J CLIN ENDOCR METAB**	41.61	10278	247	5.97	0.13
18	**ARTERIOSCL THROM VAS**	41.15	2798	68	6.37	0.08
19	**ANN INTERN MED**	41.09	3534	86	16.73	0.12
20	**OBESITY**	37.56	2404	64	4.28	0.05

* Average citations per item = Number of citations/ number of published items. Impact factor and Eigenfactor scores from Thomson Reuters JCR

## Discussion

The present study sought to provide a detailed evaluation of the T2DM published literature using large scale data, bibliometric indicators and density-equalizing mapping. A total of 24,783 items were published and cited 476,002 times over the study period. The huge number of countries included in T2DM research reflects the global burden of the disease. The density-equalizing mapping showed that the USA and the UK were responsible for the majority of the published literature. However, Switzerland received the highest citation average per item. Bilateral cooperation was the most common type of international collaboration and this was principally observed between two of the dominant countries including the USA and the UK. The leading research institutions were Harvard University in Boston, the University of California in Los Angeles and the University of London. The USA and UK also collaborated with other countries that provided a smaller research yield including Italy and Switzerland. Given the increasing prevalence of T2DM in some of the Asian countries such as India and China [29] it is vital that the leading countries make a concerted effort to share their knowledge and collaborate with these countries in future research initiatives, particularly concentrating on the management and treatment strategies for T2DM.

The quality of the research output was measured based on the average citation rate (average number of citations per item), the impact factor scores and the Eigenfactor scores. Diabetes focused journals including Diabetes, Diabetologia and Diabetes Care mainly contributed to the publication output while the New England Journal of Medicine had the highest average number of citations per item and the greatest impact factor score. The highest Eigenfactor score was awarded to the Proceedings of the National Academy of Sciences of the United States of America.

In comparison with the findings of previous studies, our results showed that research output escalated in the late 1980's, 1990's and 2000's in addition to the citation count showing the enhanced interest in T2DM research [20–23]. Specifically, articles published in 2002 received more citations (n = 38,412) than other years suggesting that there may have been more funding opportunities for research in this area given the increasing prevalence of T2DM.

The increasing research output mirrored the growing prevalence of T2DM [1]. A downward trend from 2001–2012 showed that the average number of citations per item was falling during this time perhaps due to the influx of additional published items or the citation lag associated with publications in some disease specific areas.

Strengths of this study include the use of the extensive WOS SCI-Expanded database. The database provided complete references of published items for the analysis. While PubMed would have offered similar numbers of published items, the reference data would have been incomplete. As the average number of citations per item may have been overestimated as a result of self-citation, the current study also included the impact factor and Eigenfactor scores to compare the quality of publishing journals.

There are a number of limitations to this study. The specifically developed software was an output trending and collaboration tool that was specifically designed for the WOS SCI-Expanded database so only data entries exported from this database were included in the analysis. The WOS SCI-Expanded database has access to the first available publications with archived records and would have provided a comprehensive summary of research productivity trends during the study period. The addition of the other six databases within WOS and the inclusion of databases like Scopus and PubMed would have provided a higher volume of published items and different results. For citation analysis, Scopus offers about 20% more coverage than the WOS but the WOS provides more detailed information regarding citations before 1996 [30]. Distinct from Pubmed, WOS tracks citations and has more complete data per published item i.e. authors affiliations. Author affiliations are recorded and specific information like the authors’ main organisation name, sub-organisation name(s), city, state, zone numbers and countries are documented. It would not have been possible to investigate international and institutional collaborations with Pubmed.

The search strategy may have omitted some suitable T2DM articles if the keywords, abstracts or titles of articles mentioned type 1 diabetes or gestational diabetes as all published items that focused on type 1 diabetes and gestational diabetes were excluded. In addition, some important articles may have been excluded from the analysis if the article was published in a language other than English. For example, the contribution from some countries like China and India to the body of literature was small relative to their populations. Given that translation costs may be expensive, this issue may have affected the contributions from other countries. Although this study examined the T2DM publications trends by year, country and journal, it did not investigate variables that may be associated with the output like socio-demographic and economic characteristics. This study will however contribute to the evidence base and facilitate the conduction of such studies in T2DM research. This is the first study to provide an overview of the published literature on T2DM using specifically designed software to analyse large scale data, bibliometric approaches and density-equalizing mapping.

## Conclusion

There is a rapidly growing volume of research in T2DM in parallel with the increasing prevalence of this condition globally. However, research outputs remain highly concentrated in a small number of developed countries. This has implications for research priorities globally and it is necessary to find an optimum balance between basic and applied research. There is a clear need to promote a deeper engagement through collaboration and funding mechanisms. Although the bibliometric methodology employed here has some limitations regarding the small volume of published items, we believe that these findings offer useful information to scientists and funding bodies regarding publication trends and ongoing collaborative work in T2DM research.

## Supporting Information

S1 Appendix Search Query. (DOCX)

S2 Appendix Copyright permission for Fig 2 and Fig 3. (PDF)
