# Supplementary material for: Type 2 Diabetes Research Yield, 1951-2012: Bibliometrics Analysis and Density-Equalizing Mapping
Source: PLoS One. 2015 Jul 24;10(7):e0133009. doi: 10.1371/journal.pone.0133009 (PMC4514795; doi:10.1371/journal.pone.0133009)
Supplement: S1 Appendix — (DOCX) [file pone.0133009.s001.docx]

**S1 Appendix: Search Query**

Search was conducted using WOS SCI-EXPANDED database including the topic search (TS).

1. **Type 2 Diabetes**

((NIDDM) OR (Maturity-Onset Diabetes) OR (Diabetes Mellitus Noninsulin-Dependent) OR (Diabetes Mellitus Adult-Onset) OR (Adult-Onset Diabetes Mellitus) OR (Diabetes Mellitus Adult Onset) OR (Diabetes Mellitus Ketosis-Resistant) OR (Diabetes Mellitus Ketosis Resistant) OR (Ketosis-Resistant Diabetes Mellitus) OR (Diabetes Mellitus Maturity-Onset) OR (Diabetes Mellitus Maturity Onset) OR (Diabetes Mellitus Non-Insulin Dependent) OR (Diabetes Mellitus Non-Insulin-Dependent) OR (Non-Insulin-Dependent Diabetes Mellitus) OR (Diabetes Mellitus Noninsulin Dependent) OR (Diabetes Mellitus Slow-Onset) OR (Diabetes Mellitus Slow Onset) OR (Slow-Onset Diabetes Mellitus) OR (Diabetes Mellitus Stable) OR (Stable Diabetes Mellitus) OR (Diabetes Mellitus Type II) OR (Diabetes Mellitus Type 2) OR (Maturity-Onset Diabetes Mellitus) OR (Maturity Onset Diabetes Mellitus) OR (MODY) OR (Type 2 Diabetes Mellitus) OR (Noninsulin-Dependent Diabetes Mellitus))

Records for search: 68,845

1. **Type 1 Diabetes**

((Diabetes Mellitus Insulin-Dependent) OR (Diabetes Mellitus Insulin Dependent) OR (Insulin-Dependent Diabetes Mellitus) OR (Diabetes Mellitus Insulin Dependent 1) OR (Diabetes Mellitus Juvenile-Onset) OR (Diabetes Mellitus Juvenile Onset) OR (Juvenile-Onset Diabetes Mellitus) OR (Type 1 Diabetes Mellitus) OR (Diabetes Mellitus Sudden-Onset) OR (Diabetes Mellitus Sudden Onset) OR (Mellitus Sudden-Onset Diabetes) OR (Sudden-Onset Diabetes Mellitus) OR (Diabetes Mellitus Type I) OR (IDDM) OR (Insulin-Dependent Diabetes Mellitus 1) OR (Insulin Dependent Diabetes Mellitus 1) OR (Juvenile-Onset Diabetes) OR (Diabetes Juvenile-Onset) OR (Juvenile Onset Diabetes) OR (Diabetes Mellitus Brittle) OR (Brittle Diabetes Mellitus) OR (Diabetes Mellitus Ketosis-Prone) OR (Diabetes Mellitus Ketosis Prone) OR (Ketosis-Prone Diabetes Mellitus) OR (Diabetes Autoimmune) OR (Autoimmune Diabetes))

Records for search: 71,708

1. **Diabetes, Gestational**

((Diabetes Pregnancy-Induced) OR (Diabetes Pregnancy Induced) OR (Pregnancy-Induced Diabetes) OR (Gestational Diabetes) OR (Diabetes Mellitus Gestational) OR (Gestational Diabetes Mellitus) OR (Diabetes Gestational))

Records for search: 9,229

**Final Search Query: A NOT (B OR C)**

(((NIDDM) OR (Maturity-Onset Diabetes) OR (Diabetes Mellitus Noninsulin-Dependent) OR (Diabetes Mellitus Adult-Onset) OR (Adult-Onset Diabetes Mellitus) OR (Diabetes Mellitus Adult Onset) OR (Diabetes Mellitus Ketosis-Resistant) OR (Diabetes Mellitus Ketosis Resistant) OR (Ketosis-Resistant Diabetes Mellitus) OR (Diabetes Mellitus Maturity-Onset) OR (Diabetes Mellitus Maturity Onset) OR (Diabetes Mellitus Non-Insulin Dependent) OR (Diabetes Mellitus Non-Insulin-Dependent) OR (Non-Insulin-Dependent Diabetes Mellitus) OR (Diabetes Mellitus Noninsulin Dependent) OR (Diabetes Mellitus Slow-Onset) OR (Diabetes Mellitus Slow Onset) OR (Slow-Onset Diabetes Mellitus) OR (Diabetes Mellitus Stable) OR (Stable Diabetes Mellitus) OR (Diabetes Mellitus Type II) OR (Diabetes Mellitus Type 2) OR (Maturity-Onset Diabetes Mellitus) OR (Maturity Onset Diabetes Mellitus) OR (MODY) OR (Type 2 Diabetes Mellitus) OR (Noninsulin-Dependent Diabetes Mellitus)) NOT ((Diabetes Mellitus Insulin-Dependent) OR (Diabetes Mellitus Insulin Dependent) OR (Insulin-Dependent Diabetes Mellitus) OR (Diabetes Mellitus Insulin Dependent 1) OR (Diabetes Mellitus Juvenile-Onset) OR (Diabetes Mellitus Juvenile Onset) OR (Juvenile-Onset Diabetes Mellitus) OR (Type 1 Diabetes Mellitus) OR (Diabetes Mellitus Sudden-Onset) OR (Diabetes Mellitus Sudden Onset) OR (Mellitus Sudden-Onset Diabetes) OR (Sudden-Onset Diabetes Mellitus) OR (Diabetes Mellitus Type I) OR (IDDM) OR (Insulin-Dependent Diabetes Mellitus 1) OR (Insulin Dependent Diabetes Mellitus 1) OR (Juvenile-Onset Diabetes) OR (Diabetes Juvenile-Onset) OR (Juvenile Onset Diabetes) OR (Diabetes Mellitus Brittle) OR (Brittle Diabetes Mellitus) OR (Diabetes Mellitus Ketosis-Prone) OR (Diabetes Mellitus Ketosis Prone) OR (Ketosis-Prone Diabetes Mellitus) OR (Diabetes Autoimmune) OR (Autoimmune Diabetes) OR (Diabetes Pregnancy-Induced) OR (Diabetes Pregnancy Induced) OR (Pregnancy-Induced Diabetes) OR (Gestational Diabetes) OR (Diabetes Mellitus Gestational) OR (Gestational Diabetes Mellitus) OR (Diabetes Gestational)))

Records for search: 25,271
