# Supplementary material for: Type 2 Diabetes Research Yield, 1951-2012: Bibliometrics Analysis and Density-Equalizing Mapping
Source: PLoS One. 2015 Jul 24;10(7):e0133009. doi: 10.1371/journal.pone.0133009 (PMC4514795; doi:10.1371/journal.pone.0133009)
Supplement: S2 Appendix — (PDF) [file pone.0133009.s002.pdf]

Berlin, 28th May 2015

Cristian Scutaru
Charité Universitätsmedizin Berlin
Charitéplatz 1, 10117 Berlin
Cristian.scutaru@charite.de

I grant permission to the open-access journal PLOS ONE to publish **Figure 2:Density equalizing mapping, total output by country.** Illustration of the total number of T2DM items, per country. The size of each country is scaled in proportion to the total number of publications. The colour coded legend shows the publication numbers and **Figure 3: Density equalizing mapping, average number of citations per T2DM related item by country.** The size of each country is scaled in proportion to the average number of citations per item. The colour coded legend shows the average number of citations per item. Threshold excludes countries with ≥30 items published under the Creative Commons Attribution License (CCAL) CC BY 3.0.

Cristian Scutaru
